# Guiding Ketogenic Diet with Breath Acetone Sensors

**DOI:** 10.3390/s18113655

**Published:** 2018-10-28

**Authors:** Andreas T. Güntner, Julia F. Kompalla, Henning Landis, S. Jonathan Theodore, Bettina Geidl, Noriane A. Sievi, Malcolm Kohler, Sotiris E. Pratsinis, Philipp A. Gerber

**Affiliations:** 1 Particle Technology Laboratory, Department of Mechanical and Process Engineering, ETH Zurich, CH-8092 Zurich, Switzerland; juliako@student.ethz.ch (J.F.K.); landish@student.ethz.ch (H.L.); jtheodor@student.ethz.ch (S.J.T.); sotiris.pratsinis@ptl.mavt.ethz.ch (S.E.P.); 2 Department of Endocrinology, Diabetes, and Clinical Nutrition, University Hospital Zurich, CH-8091 Zurich, Switzerland; Bettina.Geidl@usz.ch; 3 Department of Pulmonology, University Hospital Zurich, CH-8091 Zurich, Switzerland; Noriane.Sievi@usz.ch (N.A.S.); Malcolm.Kohler@usz.ch (M.K.)

**Keywords:** chemical sensor, breath analysis, semiconductor, nanotechnology, flame spray pyrolysis, point-of-care, biomedical

## Abstract

Ketogenic diet (KD; high fat, low carb) is a standard treatment for obesity, neurological diseases (e.g., refractory epilepsy) and a promising method for athletes to improve their endurance performance. Therein, the level of ketosis must be regulated tightly to ensure an effective therapy. Here, we introduce a compact and inexpensive breath sensor to monitor ketosis online and non-invasively. The sensor consists of Si-doped WO_3_ nanoparticles that detect breath acetone selectively with non-linear response characteristics in the relevant range of 1 to 66 ppm, as identified by mass spectrometry. When tested on eleven subjects (five women and six men) undergoing a 36-h KD based on the Johns Hopkins protocol, this sensor clearly recognizes the onset and progression of ketosis. This is in good agreement to capillary blood β-hydroxybutyrate (BOHB) measurements. Despite similar dieting conditions, strong inter-subject differences in ketosis dynamics were observed and correctly identified by the sensor. These even included breath acetone patterns that could be linked to low tolerance to that diet. As a result, this portable breath sensor represents an easily applicable and reliable technology to monitor KD, possibly during medical treatment of epilepsy and weight loss.

## 1. Introduction

**Figure 1:**
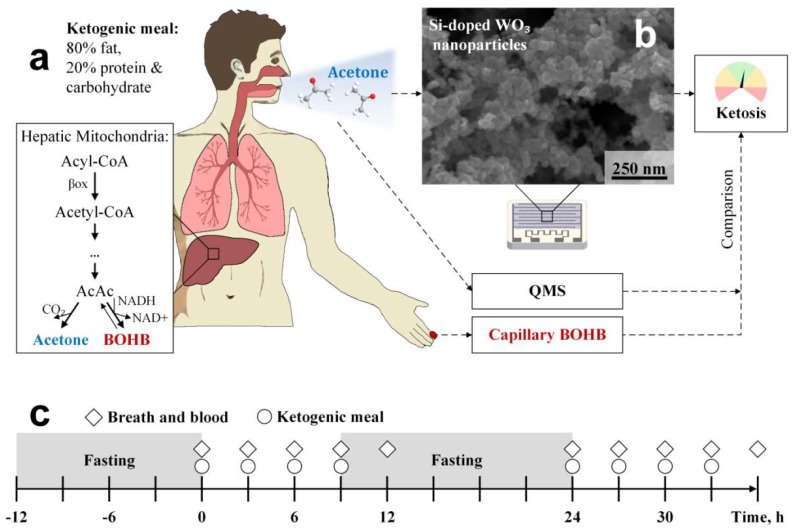
(**a**) Participants undergoing a KD feature intensified ketogenesis. Therein, acetone and BOHB are formed at elevated rates by metabolizing free fatty acids in the hepatic mitochondria (box). Acetone is volatile and can be measured non-invasively in the exhaled breath while non-volatile BOHB can be determined through capillary blood assay. To indicate the status of ketosis, breath acetone is measured with tailor-made and inexpensive chemoresistive sensing films (**b**). These consist of agglomerated and aggregated Si-doped WO_3_ nanoparticles, as indicated by SEM (top view). Sensor results are compared to parallel measurements of breath acetone by QMS and capillary blood BOHB. (**c**) Experimental protocol: Volunteers ingest a total of 8 ketogenic meals every 3 h (circles) on two consecutive days. Breath and capillary blood are analyzed 10 times, always just before the ketogenic meals and at the end of each measurement day. Prior to and in between both days, overnight fasting (gray shaded) is performed.

Ketogenic diet (KD, high fat at low carbohydrate and low protein intake) is a proven medical treatment of refractory epilepsy (i.e., drug-resistant) [1] occurring in ~30% of the approx. 50 million epileptics worldwide [2]. Also, it is an effective therapy for weight loss [3] with growing interest for treating obesity-associated metabolic disorders [4] (e.g., diabetes [5] or fatty liver disease [6]), it acts anti-inflammatory [7] and is even beneficial for athletes to improve their endurance performance by altering fuel preference [8]. During KD, body energy consumption shifts from glucose as primary fuel to the production and use of ketone bodies [9] while only moderate protein intake should limit gluconeogenesis from amino acids [10]. In specific, acetoacetate (AcAc) is such a ketone body formed in the hepatic mitochondria after β-oxidation of fatty acids and further biochemical transformations (Figure 1a, box) [9]. Its rate of production is determined also by the availability of mitochondrial 3-hydroxy-3-methyl-glutaryl-coenzyme A (HMG-CoA) synthase induced by fasting, cyclic adenosine monophosphate (cAMP) and fatty acids [11]. Other ketones are β-hydroxybutyrate (BOHB) and volatile acetone obtained from AcAc by reversible enzymatic degradation and spontaneous decarboxylation, respectively [9].

During a KD, frequent monitoring is desired to assure the persistence of the ketogenic state. Currently, this is done primarily by measuring AcAc or BOHB in urine or blood [12]. Despite blood assay’s accuracy, it is not ideal for frequent monitoring due to its invasiveness and cost. The urine assay also has drawbacks, in particular, its low precision due to varying factors such as patients’ hydration and acid-base balance [13]. Exhaled acetone measurement is a promising alternative, with breath being always accessible in a non-invasive manner [14]. In fact, measurements on 12 healthy adults revealed increasing breath acetone levels from average 0.7 to 2.5 ppm after a 12-h KD correlating well with plasma BOHB and urinary AcAc [12], a similar trend as observed in other studies [15]. This is even more pronounced in epileptic children that may follow a KD for several months, reaching breath acetone concentrations >100 ppm [16]. Most importantly, all studies reported strong inter-subject differences in ketogenic profiles at the same dietary conditions [12,15,16] highlighting the need for frequent and individual monitoring to provide personalized feed-back.

In general, the collection of repetitive measurements of biomarkers, their analysis and interpretation, serving as basis for individualized health recommendations, is considered as one of the most promising tools of modern personalized medicine. Currently, only few such systems are clinically available, but these have been shown to be superior compared to traditional systems. An example is the continuous measurement of glucose in patients with diabetes with semi-automated systems guiding insulin therapy [17].

Despite their outstanding sensitivity and selectivity, applied mass spectrometry-based methods (e.g., GC-FID [12,16], GC-MS [18], SIFT-MS [15]) are rather bulky, costly and require trained personnel impeding their application as personal breath acetone detectors in daily life. More suitable are chemical gas sensors, for instance, based on chemoresistive metal-oxides. They are applied already in indoor air monitoring with typical costs per unit of few U.S. dollars (e.g., Figaro TGS 813 for combustible gases [19]). This is enabled by scalable and CMOS compatible technologies for the sensing film fabrication. Specifically, their sensing structure can be grown [20] or deposited (e.g., by thermophoresis from flame aerosols [21] or doctor-blading [22]) directly on micro-machined chips resulting in compact sensors for ready integration into hand-held devices [23]. Furthermore, their film morphology and deposited mass can be optimized during fabrication by in-situ resistance read-out [24]. When nanostructured, such sensors exhibit high sensitivity to detect even low ppb analyte concentrations [21]. Nevertheless, selectivity against other exhaled compounds that might occur at higher concentrations remains a major challenge.

ε-phase WO_3_ nanoparticles are particularly suitable due to their outstanding selectivity (e.g., to ethanol, methanol, NO, NO_2_, CO or NH_3_) [25] and high sensitivity to detect acetone down to 20 ppb even at breath-relevant 90% relative humidity (RH) [26]. This metastable phase can be stabilized by Si- [27] or Cr-doping [25] during flame aerosol synthesis. Combined with an end-tidal breath sampler [28], such sensors have been used already to monitor fat burn rates through breath acetone online during exercise and rest in 20 volunteers in good agreement to state-of-the-art proton transfer reaction time-of-flight mass spectrometry and venous blood BOHB [29]. Even sub-ppm concentrations of breath- and skin-emitted acetone can be detected with high accuracy (i.e., 19 ppb) [30] when used in combination with isoprene-selective Ti-doped ZnO [31] and ammonia-selective Si-doped MoO_3_ [32] as an orthogonal sensor array for search and rescue applications. Note that isoprene is an attractive breath marker for physical activity [33] and has potential for non-invasive cholesterol monitoring [34] while breath ammonia could be applied to detect end-stage renal disease [35]. For KD monitoring, however, selective detection of breath acetone at higher ppm concentrations [16] is necessary. So far, Si-doped WO_3_ sensors had been characterized only up to 3 ppm at 90% RH in simulated breath [26] and up to 2.4 ppm in real breath [29].

Here, compact sensors based on Si-doped WO_3_ nanoparticles are tested to monitor ketosis during a 36-h ketogenic diet based on the Johns Hopkins protocol [36] (Figure 1). Together with a sampler for end-tidal breath extraction [28], these sensors are applied on eleven volunteers (five females and six males) to monitor their individual breath acetone profiles. Sensor responses are compared closely to quadrupole mass spectrometry (QMS) to identify the sensing characteristics at elevated breath acetone concentrations up to 66 ppm. Simultaneous capillary blood assay (BOHB and glucose) is performed as a benchmark.

## 2. Materials and Methods

### 2.1. Acetone Sensor Fabrication and Film Characterization

The sensor is based on Si-doped WO_3_ nanoparticle films prepared by flame spray pyrolysis (FSP) [26]. The precursor solution consisted of ammonium metatungstate hydrate (Sigma Aldrich, St- Louis, MO, USA, purity ≥97%) and hexamethyl disiloxane (Sigma Aldrich, purity ≥98%) diluted in a mixture of 1:1 ethanol (Sigma Aldrich, purity ≥99.8%) and diethylene glycol monobutyl ether (Sigma Aldrich, purity ≥98%) to obtain a final metal (W and Si) concentration of 0.2 M at 10 mol% Si content. This solution was fed through a capillary at 5 mL min^−1^ and dispersed by 5 L min^−1^ oxygen at a pressure drop of 1.5 bar. A surrounding premixed methane (1.25 L min^−1^)/oxygen (3.2 L min^−1^) flame was used to ignite the spray while 5 L min^−1^ sheath oxygen through an annulus surrounding the flame ensured complete combustion. Obtained nanoparticles were deposited directly by thermophoresis [37] for 4 min at 20 cm height above the burner onto interdigitated Pt electrodes (sputtered, 350 µm width and spacing) on Al_2_O_3_ substrates (15 mm × 13 mm × 0.8 mm, Electronic Design Center, Case Western Reserve University, USA). Furthermore, adhesion and cohesion were increased by in-situ annealing with a particle-free xylene-flame (11 mL min^−1^) for 30 s and an oxygen dispersion rate of 5 L min^−1^. Finally, the sensors were thermally stabilized by annealing in an oven (Carbolite Gero 30–3000 °C) at 500 °C for 5 h. The sensing film’s morphology was evaluated with a Hitachi FE-SEM 4000 scanning electron microscope (SEM) operated at 5 kV.

### 2.2. Breath and Blood Analysis

End-tidal breath was extracted in a monitored and reproducible fashion with a tailor-made and modular sampler illustrated and described in detail elsewhere (Figure 1a in ref. [28]). In brief, it consisted of an inlet to measure airway pressure and guide the exhalation flow by visual prompting and an open-ended exhalation tube to capture and buffer end-tidal breath. Volunteers were asked to exhale for 30 s through a disposable mouthpiece into the sampler while maintaining an airway pressure of 980 Pa (corresponding to 50 mL s^−1^ exhalation flow), as recommended by the American Thoracic and European Respiratory Societies for sampling of NO [38]. A CO_2_ (Capnostat 5, Respironics, Murrysville, PA, USA) was used to check if the volunteer reached the end-tidal breath portion (CO_2_ > 3% [39]) at the end of the exhalation. Breath was analyzed online by directing samples through a heated transfer line to the acetone sensor and a mass spectrometer for cross-validation. All surfaces in contact with breath consisted of inert Teflon and were heated (65 °C) to avoid water condensation and analyte adsorption.

The acetone sensor was mounted on a Macor holder, installed inside a Teflon chamber (shown in Figure 1a of [40]) and fed with 130 mL min^−1^ from the sampler with a pump (SP 135 FZ, Schwarzer Precision, Essen, Germany). For optimal selectivity and sensitivity, the acetone sensor was heated to 350 °C [40] by applying constant voltage (R&S HMC8043, HAMEG, Mainhausen, Germany) through a Pt heater located on the backside of the substrates while monitoring the temperature with a resistance temperature detector on the front. The resistance of the sensing film was continuously measured and recorded by a Multimeter (Keithley 2700, Keithley Instruments, Solon, OH, USA). The sensor response S was defined as [29]

(1) $$S=\frac{R_{air}}{R_{breath}}-1,$$

with $R_{air}$ and $R_{breath}$ being the sensor resistances in background room air and upon breath exposure, respectively.

An additional line was connected just before the acetone sensor chamber to extract samples for the QMS (QMS 422, ThermoStar^TM^, Pfeiffer Vacuum, Asslar, Germany). Secondary electron multiplier voltage was set to 950 V, while the analysis unit was heated to 150 °C. For acetone detection, the ion current at mass-to-charge ratio of 58.0 was measured with a dwell time of 0.2 s and resolution of 200. The QMS was calibrated with 9-point curves of single-component acetone in the range of 0.5 to 50 ppm in synthetic air at 90% RH. Certified acetone cylinder gas (13.6 ppm in synthetic air, Pan Gas, for concentrations ≤ 1 ppm and 500 ppm in synthetic air, Pan Gas, for concentrations > 1 ppm) was diluted in humidified synthetic air (Pan Gas 6.0, C_n_H_m_ and NO_x_ ≤ 100 ppb) with a mixing setup described elsewhere [32]. Capillary blood was sampled via finger pricking with lancet pens. BOHB and glucose were determined with a FreeStyle Neo Precision (Abbott Diabetes Care, Alameda, CA, USA).

### 2.3. Study Protocol

Healthy non-smokers free from respiratory or cardiovascular disease were included in this study. Each volunteer was informed about the experimental protocol prior to the test, gave written consent and could interrupt the test or withdraw consent anytime. This study was approved by the local ethics commission (Kantonale Ethikkommission Zürich, #2015-0675). Weight and height were measured on the first day prior to the first experiment. The participants followed a fat:(carbohydrate+protein) 4:1 KD based on the Johns Hopkins protocol [36] for 36 h including overnight fasting periods. The study timeline is illustrated schematically in Figure 1c. Each subject was asked to avoid physical exercise and alcoholic beverages 24 h before and during the experiment, fast for 12 h prior to the experiment and to avoid tooth brushing and mouth wash 2 h prior to and during the experiment to minimize exogenous confounders. Participants were allowed to drink water throughout the experiment but were asked to stop 30 min before breath and blood sampling. On the first day, each volunteer was given a total of 4 liquid ketogenic meals (circles, Figure 1c) every 3 h starting at around 8 a.m. This was followed by 15 h of fasting during the night and another 4 ketogenic meals on the second day at the same times. Breath and blood samples (diamonds, Figure 1c) were collected just before meal consumption and at the end of each testing day.

Each ketogenic meal was composed of 35% fat whipping cream (Coop, Basel, Switzerland) and chocolate-flavoured protein supplement powder (Sponsor Whey Protein 94, Wollerau, Switzerland) to accomplish a total fat content of 80 wt%. The corresponding macronutritional composition of both products is summarized in Table 1. Four meals accounted for 75% of the 24-h energy expenditure (24-EE in kcal/d) of the individual’s daily calorie requirement determined by multiplying the resting energy expenditure (REE) of each volunteer with the physical activity factor. The REE is calculated using the revised Harris-Benedict formula [41] which provides a quite accurate estimate in non-overweight subjects [42]. The physical activity factor is based on the physical activity during working and free time [43].

## 3. Results and Discussion

### 3.1. Study Cohort

Eleven volunteers (five females and six males) with a median age (±SD) of 22.7 (±2.0) years participated in this study. Their body mass index was 22.4 (±3.3) kg/m^2^. All volunteers had office jobs and were rather inactive (volunteers #1-9), moderately (volunteer #11) and highly active (volunteer #10) during their free time. Consequently, physical activity factors of 1.4, 1.5 and 1.6 were assumed, respectively, following the literature [43]. Individual physiological parameters, physical activity factors and calculated 24-EE are shown in Table 2.

### 3.2. Breath Acetone Sensor Design

The applied breath acetone sensor consisted of chemoresistive Si:WO_3_ nanoparticles with average particle size (d_BET_) of 12 nm [26]. By depositing them directly from flame-aerosols onto sensor substrates by thermophoresis [37], these nanoparticles agglomerate and aggregate to fine and porous sensing networks, as revealed by top-view SEM (Figure 1b). The open and nanostructured film morphology is advantageous for gas sensing as analytes (e.g., acetone) can access easily the sensing structure and interact with its large specific surface area. This promotes fast response times and high sensitivity to detect, for instance, 20 ppb of acetone at breath-relevant 90% RH in less than 60 s, as demonstrated on laboratory gas mixtures [40].

Human breath contains more than 870 compounds [44]. High selectivity is therefore a key requirement for a breath acetone sensor. This is addressed by employing the ferroelectric ε-phase of WO_3_ featuring a monoclinic, similar to the widely applied γ-phase, but acentric crystal configuration [45]. This metastable phase (stable below −40 °C [45]) can be stabilized at room and sensor operational temperature (350 °C [40]) by Si- [26] or Cr- [25] doping. It has been proposed that the outstanding selectivity of ε-WO_3_ is associated to its spontaneous electric dipole moment that interacts with the strong dipole moment of acetone [25]. However, also other factors may contribute to acetone selectivity, for instance, the orientation of exposed surface facets, as demonstrated with WO_3_ nanorods [46].

### 3.3. Sensor Performance and Calibration at Elevated Breath Acetone Concentrations

**Figure 2:**
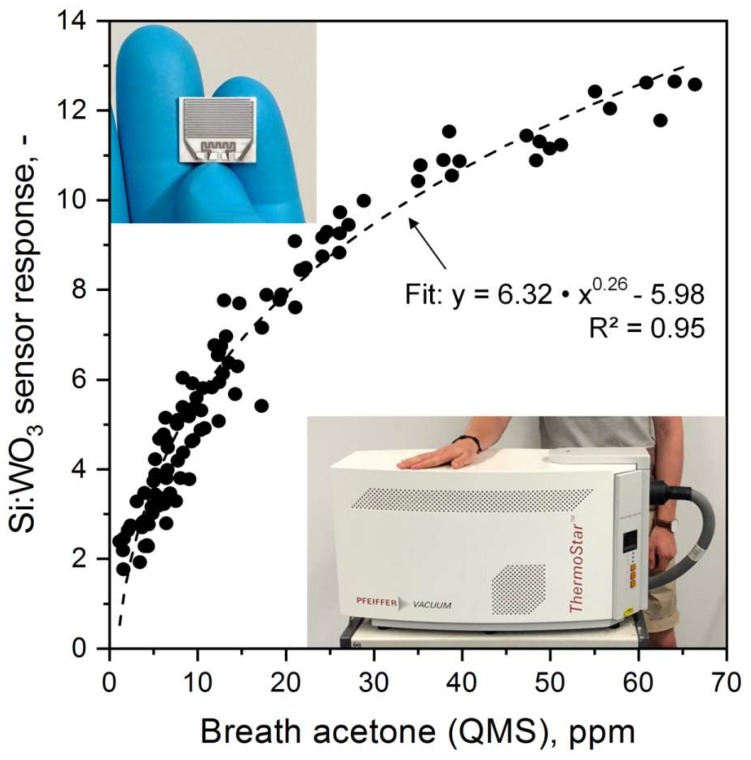
Scatter plot between the Si-doped WO_3_ sensor responses and QMS-measured acetone concentrations of 105 breath samples (11 volunteers). Power law fit is indicated as dashed line together with the corresponding coefficient of determination (R^2^). Inset images show the sensor chip (top left) and the QMS instrument (bottom right).

Si:WO_3_ sensor response and QMS-measured breath acetone concentrations from eleven volunteers (105 breath samples) from the 36-h KD are shown in Figure 2. In general, the sensor resolves the entire range from 1.1 to 66.4 ppm, a remarkable performance considering the sensor’s compact and inexpensive design compared to that of the QMS. However, the acetone sensitivity (i.e., slope of response curve) decreases with increasing breath acetone concentrations indicating non-linear response characteristics. This is similar to other flame-made metal-oxide sensors (e.g., Pt:SnO_2_ with CO [37]) at such concentrations and in agreement with non-linear diffusion-reaction theory [47]. Accordingly, the response S can be approximated with a power law [48]: $S=A\cdot c_{ace}^{B}+K$ with c_ace_ being the breath acetone concentration. Best fit (dashed line, R^2^ = 0.95) was obtained with a pre-factor A of 6.32, exponent B of 0.26 and constant K of −5.98. This transfer function was used in the following to determine breath acetone concentrations from sensor responses.

It is also worth discussing the observed spread between sensor and QMS (Figure 2). This may be related to minor interference of the sensor by other gases than acetone. In fact, Si:WO_3_ shows a smaller response, for instance, to ethanol [26] or isoprene [40] that are contained typically at sub-ppm concentrations in sober and healthy breath [49,50]. Also the high relative humidity in exhaled breath alters the sensor response to acetone [26]. To compensate for these, the Si:WO_3_ sensor could be combined with other selective sensors (e.g., Ti-doped ZnO for isoprene [31], Si-doped MoO_3_ for ammonia [32] and commercial humidity sensors) in orthogonal arrays [30]. Such improved accuracy is especially required when lower ppb concentrations of acetone need to be detected (e.g., in search and rescue when sniffing entrapped humans [30]) but less important in KD monitoring where exhaled acetone concentrations are at elevated ppm levels (Figure 2). Also filters (e.g., microporous membranes [51] for larger interfering molecules or adsorption packed beds [52]) or GC columns [53] are quite effective to improve selectivity.

### 3.4. Monitoring Individual Ketosis through Breath and Blood

**Figure 3:**
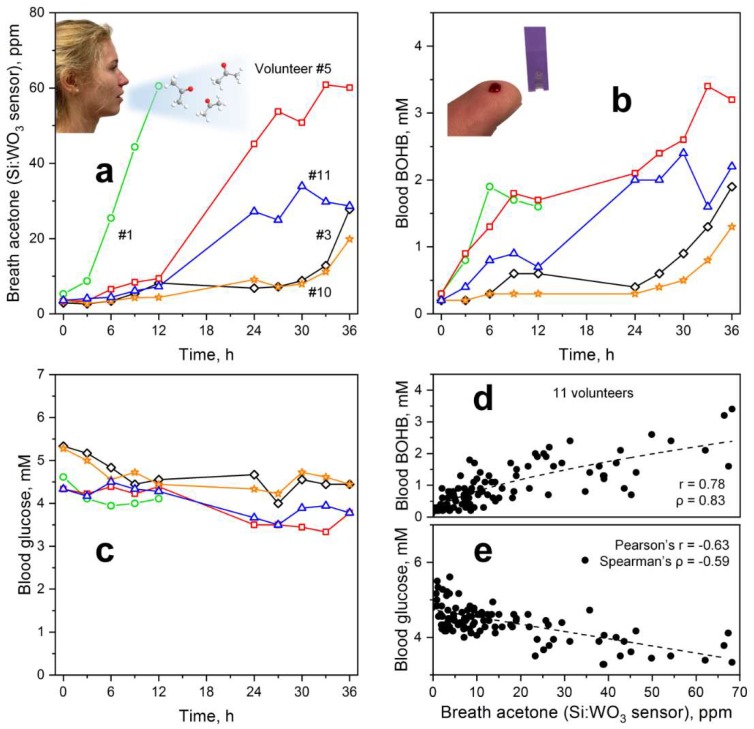
Individual (**a**) breath acetone levels as determined by the Si-doped WO_3_ sensor, capillary blood (**b**) BOHB and (**c**) glucose concentrations of five representative volunteers during a 36-h KD. Note that volunteer #1 (green circles) had to abort the experiment already after 24 h due to strong nausea. Scatter plot of (**d**) BOHB and (**e**) glucose versus acetone concentrations for all eleven volunteers (105 samples) with corresponding Pearson’s (r) and Spearman’s (ρ) correlation coefficients. Dashed lines indicate fitted (power law in d and linear in e) trend lines.

As a next step, the individual sensor-measured breath acetone dynamics of the volunteers during the KD were analyzed and compared to a commercial capillary blood BOHB and glucose monitoring system. Figure 3 shows the simultaneously measured profiles for (a) breath acetone, (b) capillary blood BOHB and (c) glucose of five representative volunteers during a 36-h KD. Data of all eleven volunteers are provided in Figure S1. For volunteer #3 (black diamonds), breath acetone almost tripled within the first 12 h, similar to other KD studies where an average increase by a factor of 3.5 was observed [12]. Most interestingly, the strongest increase was observed after 30 h when breath acetone concentrations exceeded 20 ppm, which should reflect advanced ketosis from intensified ketogenesis [9] (Figure 1a, box). This is significantly higher than observed, for instance, during exercise and post-exercise rest where breath acetone levels did not exceed 3 ppm [29]. Remarkably, capillary blood BOHB (Figure 3b) as an established marker for ketosis followed the same dynamic. In specific, nutritional ketosis (0.5–3 mM [54]) is entered after 9 h and mild ketosis (2–7 mM [55]) after 36 h of KD, the latter is needed for an efficient treatment of epilepsy [56]. This indicates that the present breath acetone sensor is suitable to monitor ketosis during KD and, most importantly, it operates non-invasively.

Within the first 12 h, a similar breath acetone trend was observed also for volunteers #5 (red squares), #10 (orange stars) and #11 (blue triangles). However, #5 and #11 differed significantly afterwards. In particular, breath acetone concentrations of both increased strongly already during overnight fasting (*t* = 12–24 h). On the second day, further increase was observed for volunteer #5 who reached breath acetone concentrations above 60 ppm after 33 h, while they leveled off for #11 at around 30 ppm. These breath acetone trends were in agreement with BOHB (Figure 3b) where volunteer #5 reached highest levels above 3 mM toward the end of the KD. It is also interesting to observe that BOHB increased stronger than the breath acetone levels within the first 12 h for volunteer #11 and especially #5. This may be related to the dynamic equilibrium between BOHB and AcAc (Figure 1a, box) [9]. In summary, the volunteers showed distinctly different ketosis dynamics despite *similar* KD conditions, as recognized correctly by the sensor.

Finally, it is worth discussing volunteer #1 (Figure 3a, green circles) who showed a distinctly different breath acetone profile. Most notably, this volunteer had already an extraordinary increase in breath acetone at the beginning of the KD reaching about 60 ppm after 12 h. A similar trend within the first 6 h is observed for blood BOHB (Figure 3b), though less distinct from the other volunteers (e.g., #5, red squares) than observed for breath acetone (Figure 3a). Interestingly, BOHB decreases thereafter. Glucose concentrations (Figure 3c, green circles), on the other hand, decreased until *t* = 6 h and started to increase slightly thereafter coinciding with the drop in BOHB levels. On the second day, this volunteer had to stop the KD due to strong nausea. This is probably caused by ketones activating the chemoreceptor zone in the vomiting center of the brain that is a known reason for nausea and vomiting in ketosis [57]. As a result, this volunteer showed low tolerance to the KD protocol that was reflected in an abnormal breath acetone pattern. In a next step, the present breath acetone sensor could be used to guide this volunteer to an optimized KD protocol (e.g., different nutritional composition) to achieve and maintain a healthy status of ketosis.

Corresponding capillary glucose levels (Figure 3c) of the five volunteers (for all volunteers, see Figure S1c) were consistently below 5.5 mM at the start and during the KD. This suggests that they adhered to the overnight fasting prior to the KD and stayed abstinent from other external carbohydrate sources during the KD (see Figure 1c for protocol). During the KD, glucose levels typically decreased within the first 12 h and leveled off thereafter between 3 and 5 mM probably due to gluconeogenesis to inhibit hypoglycemia [10], as suggested above.

### 3.5. Correlations between Breath and Blood Parameters

The blood and breath ketone concentrations for all volunteers are shown in Figure 3d. Breath acetone and capillary blood BOHB correlated significantly with Spearman’s rank correlation coefficient ρ of 0.83 (*p* < 0.001) and Pearson’s correlation coefficient r of 0.78 (*p* < 0.001), as expected from their similar intra-subject dynamics during KD (Figure 3a vs. Figure 3b) and joint biochemical origin as products of ketogenesis (Figure 1a, box) [9]. When applying a power law fit (dashed line), a degree of determination (R^2^) of 0.62 is obtained that is well within the range of fasting and dieting experiments (0.54 ≤ R^2^ ≤ 0.94) [14].

A weaker and *inverse* correlation was found between breath acetone and capillary blood glucose (r = −0.63, ρ = −0.59, both *p* < 0.001). This was anticipated from the observed trends during KD (Figure 3a vs. Figure 3c). Ketogenesis is driven by hepatic β-oxidation which increases during fasting when lipolysis increases as no longer suppressed by insulin and by the expression of mitochondrial HMG-CoA synthase which is induced by fasting and downregulated by insulin [11]. Although low insulin and high glucagon levels resulting from low blood glucose serve as an initial trigger for ketogenesis, ketone body levels are rather determined by glucose level-independent effects, such as free fatty acid availability for ketone body production and ketone body consumption by peripheral tissues [58].

## 4. Conclusions

A compact and inexpensive breath acetone sensor was introduced to monitor the status of ketosis during ketogenic diets. Its sensing film consisted of chemoresistive Si-doped WO_3_ nanoparticles that showed high selectivity and non-linear response characteristics for breath acetone concentrations up to around 66 ppm, as revealed by mass spectrometry. When applied on eleven volunteers during a 36-h ketogenic diet, the breath sensor accurately followed the individual breath acetone dynamics, in general agreement with capillary blood BOHB. Most interestingly, strong differences in individual breath and blood profiles were identified between volunteers despite identical KD conditions. These were correctly recognized by the breath acetone sensor including patterns possibly associated to low tolerance to the KD protocol. The sensor’s ability to accurately and rapidly monitor individual changes in the ketogenic state together with its compact size renders it attractive for repetitive measurements by the public. As a result, this breath sensor is promising as a portable ketosis monitor in a broad range of applications including personalized, non-pharmaceutical treatment of epileptics and guiding of dietary interventions for weight loss.

## 5. Patents

A.T.G. and S.E.P. declare a patent application.

## Figures and Tables

**Table 1:** Macronutrient composition of ketogenic meals.

Macronutrient	Whipping Cream [wt%]	Protein Supplement Powder [wt%]
Fat	35	2
Carbohydrates	3	3.8
Protein	3	80
Calories [kcal/g]	3.37	3.65

**Table 2:** Demographic and anthropometric data as well as calculated energy expenditures of the participants.

Volunteer [-]	Gender [-]	Age [y]	Weight [kg]	Height [m]	BMI [kg/m²]	Physical Activity Factors [-]	24-h Energy Expenditure [kcal/d]
1	f	21	58.0	1.59	22.94	1.4	1831
2	m	23	69.4	1.77	22.15	1.4	2352
3	m	22	72.0	1.80	22.22	1.4	2610
4	f	22	51.4	1.63	19.35	1.4	1753
5	m	22	51.8	1.78	16.35	1.4	1598
6	f	28	78.8	1.63	29.66	1.4	2234
7	f	25	74.1	1.71	25.34	1.4	2142
8	m	22	73.2	1.73	24.46	1.4	1807
9	m	22	74.5	1.80	22.99	1.4	2471
10	m	22	70.8	1.82	21.37	1.6	2786
11	f	21	59.4	1.72	20.08	1.5	2100

## Supplementary Materials

The following are available online at http://www.mdpi.com/1424-8220/18/11/3655/s1, Figure S1: Individual (a) breath acetone, capillary blood (b) BOHB and (c) glucose levels of 11 volunteers during a 36-h ketogenic diet. Note that volunteer #1 (green circles) aborted the experiment already after 24 h due to strong nausea. Volunteer #4 (pink crosses) suffered also from nausea after the overnight sleep (approx. at t = 22 h) and took a small dose of dextrose resulting in the observed blood glucose peak at t = 24 h.
