# Supplementary material for: Guiding Ketogenic Diet with Breath Acetone Sensors
Source: Sensors (Basel). 2018 Oct 28;18(11):3655. doi: 10.3390/s18113655 (PMC6264102; doi:10.3390/s18113655)
Supplement: Supplementary file 1 [file sensors-18-03655-s001.pdf]

# Supplementary Information

## Guiding ketogenic diet with breath acetone sensors

Andreas T. Güntner[1*], Julia F. Kompalla[1], Henning Landis[1], S. Jonathan Theodore[1], Bettina Geidl[2], Noriane A. Sievi[3], Malcolm Kohler[3], Sotiris E. Pratsinis[1] and Philipp A. Gerber[2*]

[1] Particle Technology Laboratory, Department of Mechanical and Process Engineering, ETH Zurich, CH-8092 Zurich, Switzerland
[2] Department of Endocrinology, Diabetes, and Clinical Nutrition, University Hospital Zurich, CH-8091 Zurich, Switzerland
[3] Department of Pulmonology, University Hospital Zurich, CH-8091 Zurich, Switzerland

*corresponding authors: Andreas.Guentner@ptl.mavt.ethz.ch
Philipp.Gerber@usz.ch

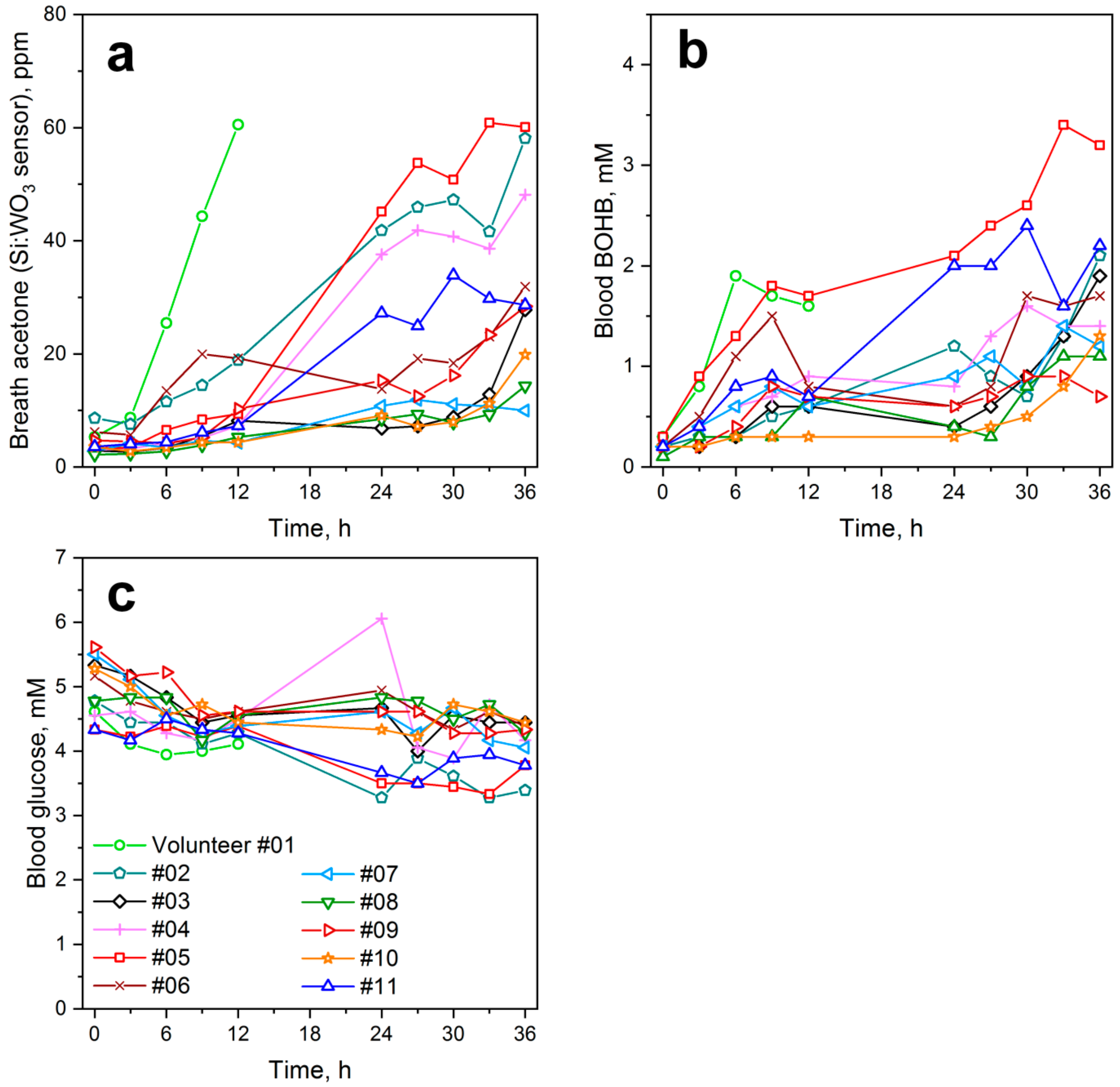


**Figure S1:** Individual (a) breath acetone, capillary blood (b) BOHB and (c) glucose levels of 11 volunteers during a 36-h ketogenic diet. Note that volunteer #1 (green circles) aborted the experiment already after 24 h due to strong nausea. Volunteer #4 (pink crosses) suffered also from nausea after the overnight sleep (approx. at t = 22 h) and took a small dose of dextrose resulting in the observed blood glucose peak at t = 24 h.
